# The integrated incretin effect is reduced by both glucose intolerance and obesity in Japanese subjects

**DOI:** 10.3389/fendo.2024.1301352

**Published:** 2024-06-20

**Authors:** Akihiro Hamasaki, Norio Harada, Atsushi Muraoka, Shunsuke Yamane, Erina Joo, Kazuyo Suzuki, Nobuya Inagaki

**Affiliations:** ^1^ Department of Diabetes, Endocrinology and Nutrition, Graduate School of Medicine, Kyoto University, Kyoto, Japan; ^2^ Department of Diabetes and Endocrinology, Medical Research Institute Kitano Hospital, PIIF Tazuke-Kofukai, Osaka, Japan

**Keywords:** incretins, gastric inhibitory polypeptide/glucose-dependent insulinotropic polypeptide, glucagon-like peptide-1, insulin, glucagon

## Abstract

**Introduction:**

Incretin-based drugs are extensively utilized in the treatment of type 2 diabetes (T2D), with remarkable clinical efficacy. These drugs were developed based on findings that the incretin effect is reduced in T2D. The incretin effect in East Asians, whose pancreatic β-cell function is more vulnerable than that in Caucasians, however, has not been fully examined. In this study, we investigated the effects of incretin in Japanese subjects.

**Methods:**

A total of 28 Japanese subjects (14 with normal glucose tolerance [NGT], 6 with impaired glucose tolerance, and 8 with T2D) were enrolled. Isoglycemic oral (75 g glucose tolerance test) and intravenous glucose were administered. The numerical incretin effect and gastrointestinally-mediated glucose disposal (GIGD) were calculated by measuring the plasma glucose and entero-pancreatic hormone concentrations.

**Results and discussion:**

The difference in the numerical incretin effect among the groups was relatively small. The numerical incretin effect significantly negatively correlated with the body mass index (BMI). GIGD was significantly lower in participants with T2D than in those with NGT, and significantly negatively correlated with the area under the curve (AUC)-glucose, BMI, and AUC-glucagon. Incretin concentrations did not differ significantly among the groups. We demonstrate that in Japanese subjects, obesity has a greater effect than glucose tolerance on the numerical incretin effect, whereas GIGD is diminished in individuals with both glucose intolerance and obesity. These findings indicate variances as well as commonalities between East Asians and Caucasians in the manifestation of incretin effects on pancreatic β-cell function and the integrated capacity to handle glucose.

## Introduction

Insulin secretion is greater with oral glucose loading than with intravenous glucose loading under the same blood glucose concentration increase (1, 2). This phenomenon is due to intestinal-derived factors released from the gut after glucose ingestion and enhanced insulin secretion from pancreatic β-cells. The intestinal tract-derived factors are called incretins, and their insulinotropic effect of them is called the incretin effect (3). Glucose-dependent insulinotropic polypeptide (GIP) and glucagon-like polypeptide-1 (GLP-1) are known as incretins (4, 5). Both GIP and GLP-1 bind to their specific receptors, GIPR and GLP1R, respectively, and stimulate insulin secretion in pancreatic β cells depending on the glucose (4, 5). GIP and GLP-1, however, are quickly cleaved the two NH2-terminal amino acids by the degrading enzyme dipeptidyl peptidase-4 (DPP-4) and inactivated (4).

The incretin effect is attenuated with a deterioration in glucose tolerance, resulting in a diminished insulin response to oral glucose loading. The incretin effect is clearly lower in type 2 diabetes (T2D) than in normal glucose tolerance (NGT) (6–11). Therefore, various strategies to enhance the incretin effect have been developed for T2D treatment (4, 12–15). Treatment that inhibit DPP-4 enzymatic activity to prolong the presence of active incretins in the plasma, and the use of a GLP1R agonist resistant to DPP4 degradation are successfully applied throughout the world (4, 12, 13, 16–18). Previous findings indicate that enhanced insulin secretion and suppression of inappropriate glucagon secretion are the major clinical effects of incretin-based drugs (19, 20). Both glucose intolerance and obesity attenuate the incretin effect and the effectiveness of incretin-related drugs (8–11).

The findings of diminished incretin effects due to impaired glucose tolerance are mainly based on studies in Caucasians (8–11), while in a study of East Asians with a background of weak β-cell function, the incretin effect in patients with T2D was comparable to that in subjects with NGT (21). These findings suggest that racial differences affect the relationship between glucose tolerance and the incretin effect, but this has not been fully investigated. Furthermore, the adequacy of the incretin effect for capturing the capacity for glucose metabolism varies (12, 22); for example, the numerical incretin effect evaluated by insulin secretion does not differ before and after DPP-4 inhibitor treatment in patients with T2D (23, 24). Gastrointestinally-mediated glucose disposal (GIGD), evaluated as the amount of intravenously infused glucose that reproduces the blood glucose excursion following oral loading, however, is improved by DPP-4 inhibitor treatment (23, 24). Based on these findings, it is important to clarify what the incretin effect actually represents (12, 25). To date, only a few investigators have examined factors other than the relationship between the incretin effect and insulin secretion.

In the present study, we investigated the relationship of glucose tolerance and obesity with the numerical incretin effect in East Asians. In addition, we examined the characteristics of GIGD as a more widely defined incretin effect in relation to glucose tolerance and obesity.

## Methods

This study protocol complied with the principles of the Declaration of Helsinki and was approved by the ethics committee of Kyoto University (registration no. C-0352). Written informed consent was obtained from all participants.

### Subjects

Twenty-eight Japanese subjects (14 with NGT, 6 with impaired glucose tolerance (IGT), and 8 with T2D) were enrolled. Diagnosis of NGT, IGT and T2D was made according to the criteria set by the Japan Diabetes Society (26). None of the participants with T2D took antidiabetic drugs.

### Procedures

Participants underwent an oral glucose tolerance test (OGTT) and an isoglycemic intravenous glucose infusion (IIGI) after an overnight fast on separate days. A standard OGTT with 75 g glucose was performed over 180 min. In the IIGI, an intravenous glucose infusion (20% wt/vol) was performed aiming to reproduce the plasma glucose profile of the OGTT using an artificial pancreas system (STG-22; Nikkiso, Tokyo, Japan). The glucose infusion rate was regulated using a previously reported glucose clamp protocol (27) with some modifications. Blood samples were collected at -15, 0, 10, 20, 30, 60, 90, 120, 150, and 180 min after starting the glucose loading, and then centrifuged at 1880 g for 10 min at 4°C. The sample supernatant was collected, and the plasma samples were stored at -80°C until analyzed.

### Analyses

The plasma glucose (PG) levels were measured by the ultraviolet absorption spectrophotometry method. Serum immunoreactive insulin (IRI) levels and C-peptide immunoreactivity (CPR) were measured by 2 -site radioimmunoassay. Plasma glucagon concentrations were determined by radioimmunoassay (Millipore, Billerica, MA, USA). Total gastric inhibitory peptide (GIP) and GLP-1 concentrations were evaluated using a human total GIP enzyme-linked immunosorbent assay (ELISA) kit (Linco Research, St Charles, MO, USA) and human total GLP-1 ELISA kit (Meso Scale Discovery, Gaithersburg, MD, USA), respectively as previously described (28, 29). After solid phase extraction (30, 31), the concentration of GIP and GLP-1 in their active forms ware measured using a human active GIP ELISA kit (IBL Co Ltd, Fujioka, Japan) and a human active GLP-1 ELISA kit (Millipore, Billerica, MA, USA), respectively.

### Calculations and statistical analyses

Areas under the curve (AUCs) were calculated using the trapezoidal rule. Incretin effect values were calculated in relation to the difference in the integrated incremental β-cell secretory responses (iAUC: baseline subtracted from the actual values) according to the following formula: 100% x (iAUC-OGTT – iAUC-IIGI)/iAUC-OGTT. The ratio of the difference in glucose administration between oral and intravenous glucose administration for 75 g oral glucose loading was calculated as the GIGD according to the following formula: 100% x (glucose-OGTT (75 g) – glucose-IIGI)/glucose-OGTT (75 g) (6, 22). As representative indices of the insulin secretion capacity and insulin sensitivity, the insulinogenic index (32, 33) and assessment of insulin resistance by homeostatic model assessment (HOMA-IR) (34, 35), respectively, were calculated as follows: insulinogenic index = (IRI at 30 min – IRI at 0 min [pM])/(PG at 30 min – PG at 0 min [mM]), HOMA-IR = (IRI at 0 min [pM]) x (PG at 0 min [mM])/22.5. Data are presented as mean ± standard deviation. Data were analyzed using a 2-tailed t-test, Wilcoxon signed rank sum test, and Kruskal-Wallis test procedures. Statistical analyses were performed with EZR (Saitama Medical Center, Jichi Medical University, Saitama, Japan), which is a graphical user interface for R (The R Foundation for Statistical Computing, Vienna, Austria) (36).

## Results

### Study participant characteristics, glucose tolerance and incretin concentrations

**Figure 1:**
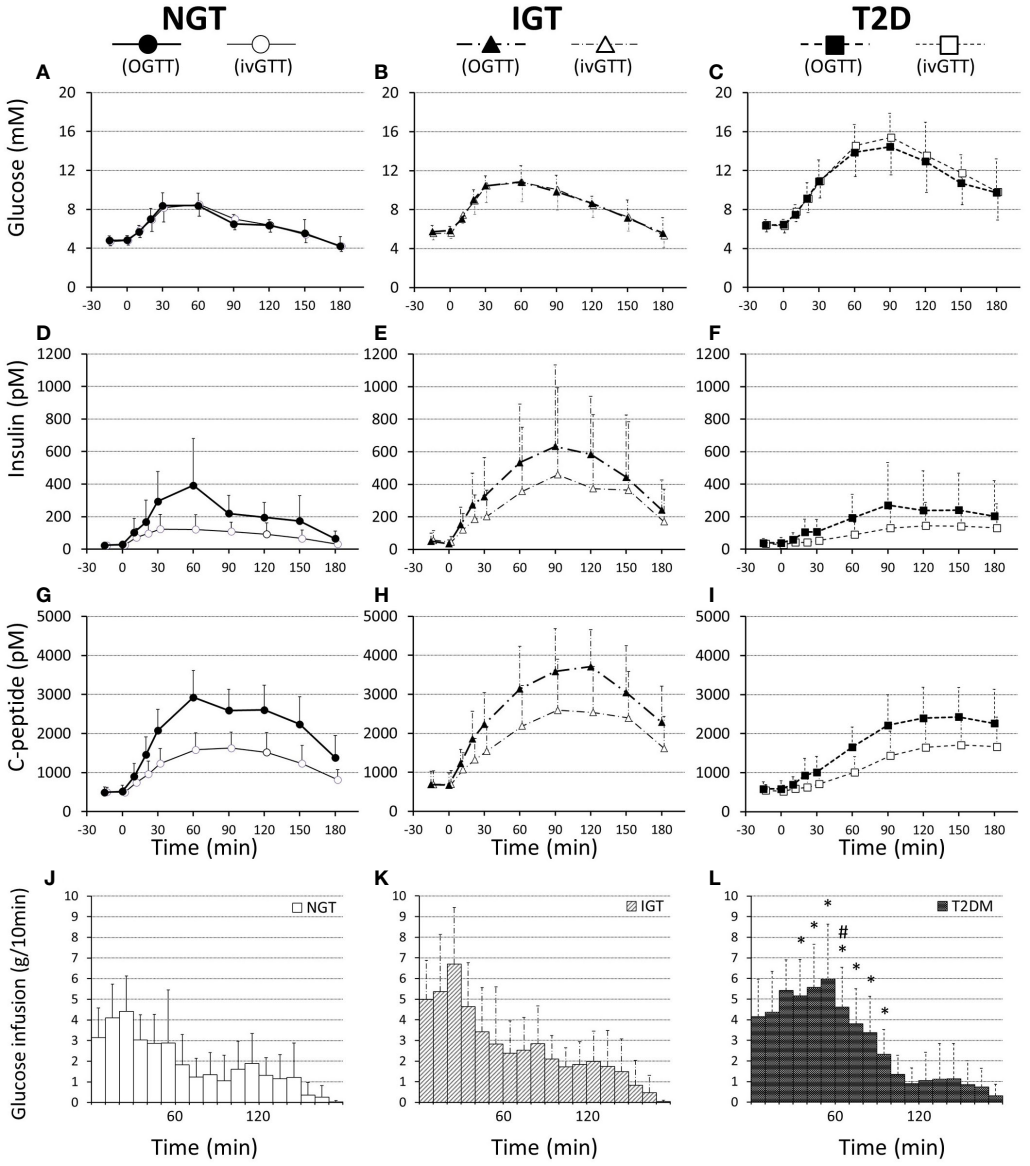
Plasma glucose **(A–C)**, insulin **(D–F)**, and C-peptide **(G–I)** concentrations and infused glucose amounts **(J–L)** during 75-g OGTT and isoglycemic intravenous glucose infusion, respectively, in participants with NGT **(A, D, G, J)**, IGT **(B, E, H, K)**, and T2D **(C, F, I, L)**. Data are mean values ± SD. * and # represent a statistical difference vs NGT and IGT, respectively (*p < 0.05*).

**Figure 2:**
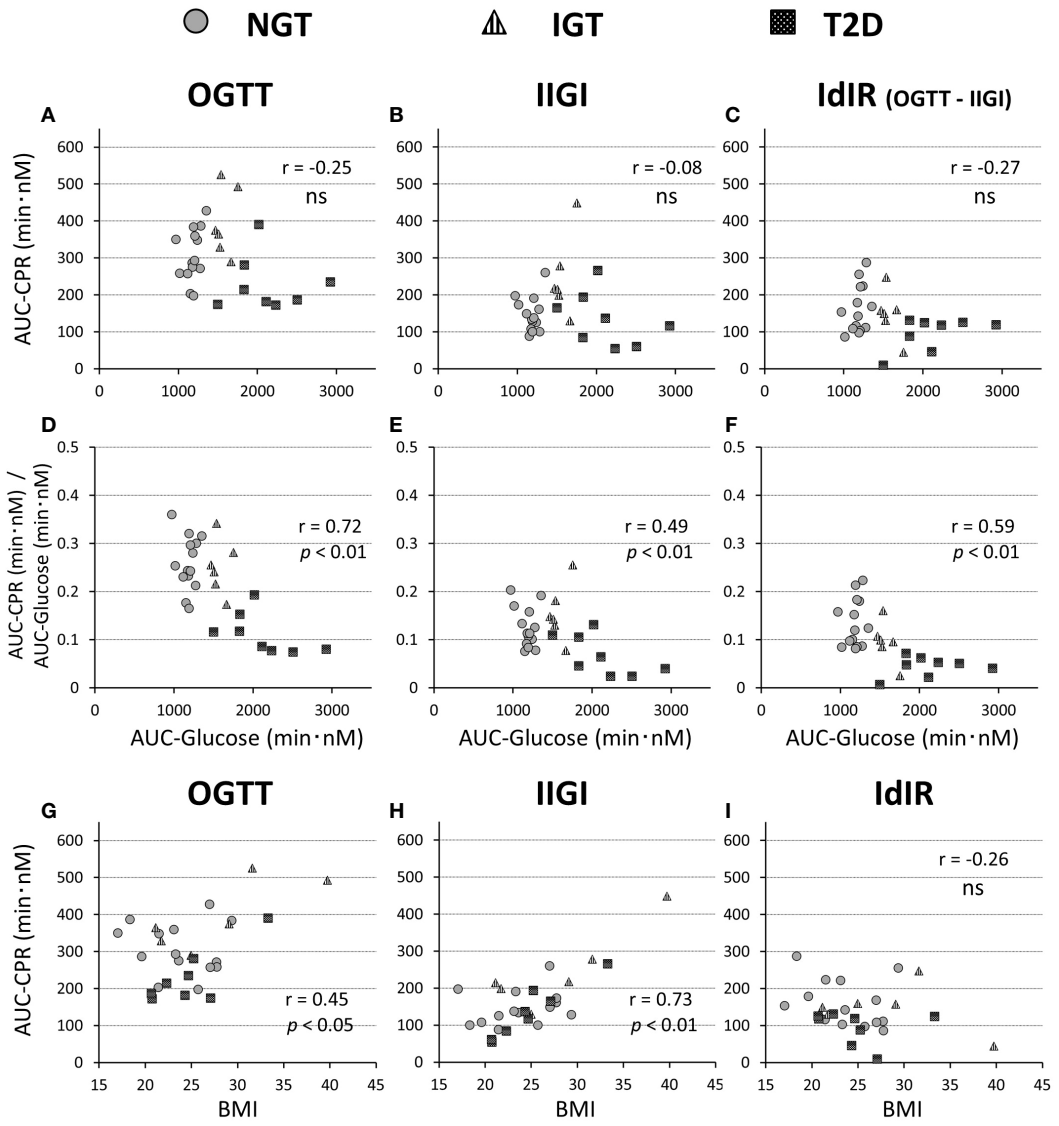
Relationships of the AUC of C-peptide values during a 75-g OGTT **(A, G)**, isoglycemic intravenous glucose infusion (IIGI) **(B, H)**, and the intestinal factors derived C-peptide(insulin) response (IdIR) (calculated by AUC of C-peptide [OGTT] – AUC of C-peptide [IIGI]) **(C, I)**, with AUC of glucose concentration during OGTT **(A–C)** and BMI **(G–I)**, respectively. Relationships of the ratios of C-peptide amounts in OGTT **(D)**, IIGI **(E)**, and IdIR **(F)** to the glucose concentration during OGTT **(D–F)** with the AUC of glucose concentrations. Pearson’s correlation tests were carried out to calculate the correlation coefficient (r) and p-values. 'ns' indicates 'not significant' (P>=0.05).

The characteristics of the study participants are shown in **Table 1**. The mean age of participants in the T2DM and IGT groups was significantly higher than that of participants in the NGT group. The mean BMI did not differ significantly among groups. Fasting blood glucose and HbA1c levels increased along with deterioration of glucose tolerance (**Table 1**). Fasting IRI, CPR, and glucagon levels did not differ significantly among the glucose tolerance groups, although the insulinogenic index level was characteristically lower in the T2D group (**Table 1**). From NGT to IGT and T2D, glucose excursions during OGTT increased, and the effect was well reproduced by IIGI (**Figures 1A–C**; **Table 2**). The IRI and CPR concentrations in the early phase after both oral and intraveneous glucose loading were lower in the T2D group compared with the other groups (**Figures 1D–I**). The insulin secretory response to glucose by AUC-CPR per glucose excursion (AUC-CPR/AUC-glucose) revealed that insulin secretion clearly decreased along with the deterioration of glucose tolerance (**Figure 2**).

**Table 1:** Participant characteristics.

	NGT	IGT	T2D
N (female)	14(3)	6(0)	8(1)
Age (years)	31.9±7.4	45.7±11.6 *	62.1±5.4 *^,#^
BMI	23.8±3.8	28.0±8.0	24.8±4.1
HbA1c (%)	5.1±0.2	5.4±0.3	6.8±0.9 *^,#^
fPG (mM)	4.8±0.4	5.9±0.4 *	6.5±0.5 *
fIRI (pM)	28.8±18.0	34.3±24.5	39.3±32.8
fCPR (pM)	514±159	673±310	585±205
fGlucagon (pM)	28.4±12.2	39.0±21.0	22.9±6.5
HOMA-IR	1.0±0.7	1.5±1.1	1.8±1.4
Insulinogenic index	73±42	68±65	16±11 *

Subjects with normal glucose tolerance (NGT), patients with impaired glucose tolerance (IGT) and type 2 diabetes mellitus (T2D). Data are mean values ± SD. * and # represent statistical differences NGT and IGT, respectively *(p < 0.05)*. BMI, body mass index; HbA1c, glycated hemoglobin AIC; f, fasting; PG, plasma glucose; IRI, immunoreactive insulin; CPR, c-peptide immunoreactivity; HOMA-IR, homeostasis model assessment of insulin resistance.

**Table 2:** Responses of glucose, insulin and C-peptide during OGTT and IIGI, and Incretin effect.

	NGT (n=14)	IGT (n=6)	T2D (n=8)
AUC-Glucose (min·mM)			
** **OGTT	1184±101	1576±109 *	2121±443 *^,#^
** **IIGI	1197±88	1577±182 *	2223±486 *^,#^
** *P*** (OGTT vs IIGI)	ns	ns	ns
AUC-Insulin (min·nM)			
** **OGTT	39.1±21.5	80.4±47.1	35.3±31.4 ^#^
** **IIGI	16.3±10.3	56.5±61.9 *	19.2±16.8
** *P*** (OGTT vs IIGI)	<0.01	ns	<0.01
AUC-CPR (min·nM)			
** **OGTT	399±84	517±134	334±104 ^#^
** **IIGI	235±63	374±168 *	228±94
** *P*** (OGTT vs IIGI)	<0.01	<0.05	<0.01
Incretin Effect (%)			
** **Insulin	61.9±15.6	44.0±30.2	51.8±21.4
** **CPR	51.5±12.9	39.0±15.8	42.6±22.8
Glucose Infusion			
** **Glucose (g)	33.7±11.9	47.9±13.7	52.2±15.2 *
** **GIGD (%)	55.1±15.8	36.1±18.2	30.4±20.3 *

Area under the curve (AUC) of plasma glucose, insulin, and C-peptide during 75g oral glucose tolerance test (OGTT) and isoglycemic intravenous glucose infusion (IIGI). Incretin effect calculated by incremental insulin and C-peptide responses during glucose load (numerical incretin effect). Gastrointestinally mediated glucose disposal (GIGD) was calculated by total amounts of glucose infused during I IGT to reproduce glucose excursion in 75g OGTT. Data are mean values ± SD. * and # represent statistical difference vs NGT and IGT, respectively *(p < 0.05)*.

'ns' indicates 'not significant' (P>=0.05).

**Figure 3:**
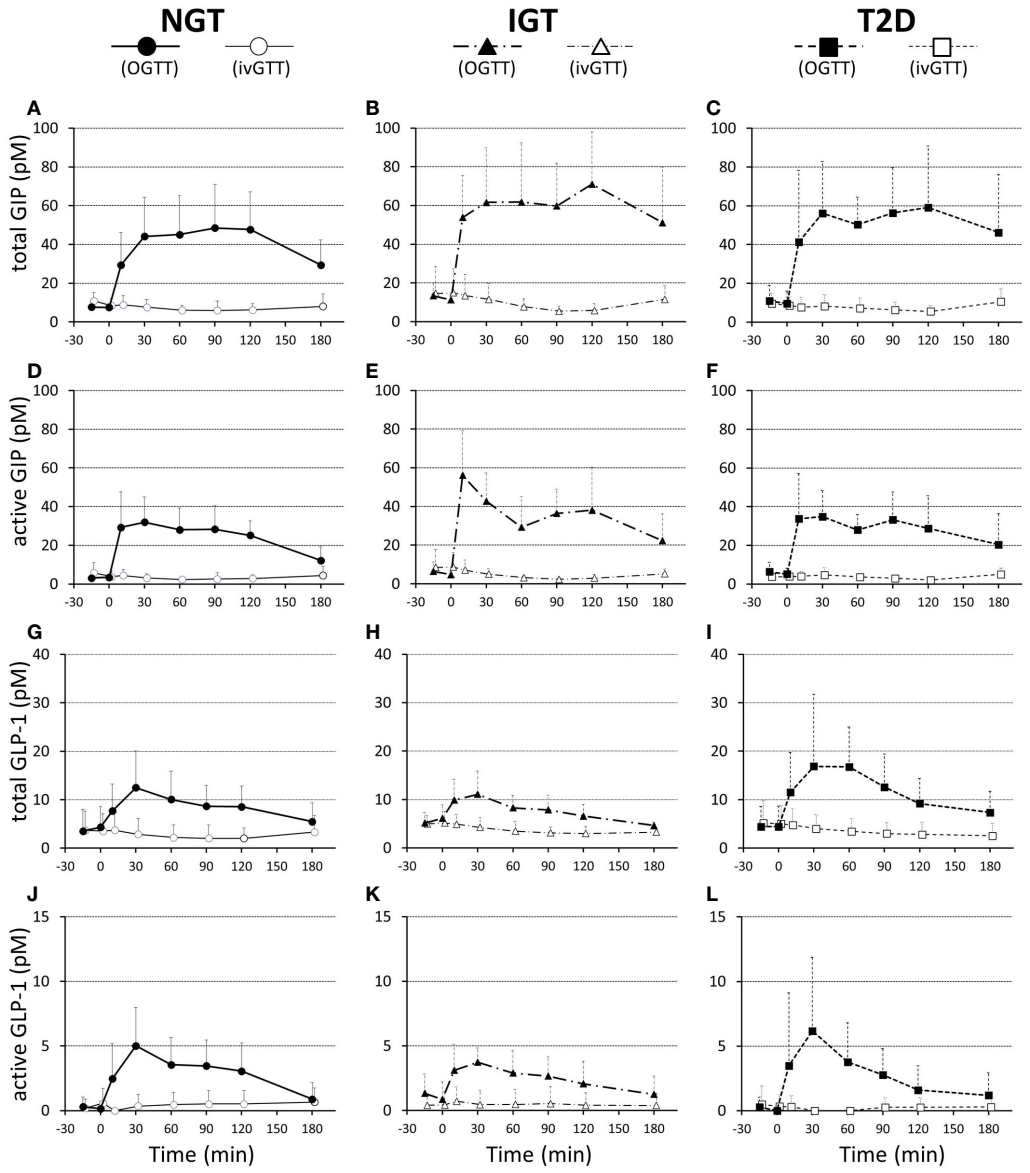
Plasma total GIP **(A–C)**, active GIP **(D–F)**, total GLP-1 **(G–I)**, and active GLP-1 **(J–L)** concentrations during 75-g OGTT and isoglycemic intravenous glucose infusion (ivGTT) in participants with NGT **(A, D, G, J)**, IGT **(B, E, H, K)**, and T2D **(C, F, I, L)**. Data are mean values ± SD.

Both GIP and GLP-1 were well secreted after oral glucose loading, while secretion was not evoked during IIGI (**Figure 3**; **Table 3**). Analysis in all 28 cases revealed that total GIP, active GIP and total GLP-1 concentrations were significantly suppressed after intravenous glucose loading (total GIP *p* = 0.010, active GIP *p* = 0.0032, total GLP-1 *p* = 0.036). Neither secretion of total GIP and GLP-1 nor enhancement of active GIP and GLP-1 during glucose loading differed significantly among groups (**Table 3**). AUC-total GIP significantly positively correlated with the BMI (r = 0.39, *p = 0.043*).

**Table 3:** Responses of plasma GIP, GLP-I, and glucagon during OGTT and IIGI.

	NGT (n=14)	IGT (n=6)	T2D (n=8)
AUC-total GIP (min·nM)			
** **OGTT	7.4±2.9	10.8±4.2	9.3±4.4
** **IIGI	1.3±0.5	1.6±0.8	1.3±0.8
** *P*** (OGTT vs IIGI)	<0.01	<0.05	<0.01
AUC-active GIP (min·nM)			
** **OGTT	4.4±1.2	6.3±2.5	5.2±2.1
** **IIGI	0.6±0.3	0.7±0.3	0.6±0.3
** *P*** (OGTT vs IIGI)	<0.01	<0.05	<0.01
AUC-total GLP-1 (min·nM)			
** **OGTT	1.6±0.8	1.4±0.3	2.1±1.1
** **IIGI	0.5±0.5	0.6±0.3	0.6±0.5
** *P*** (OGTT vs IIGI)	<0.01	<0.05	<0.01
AUC-active GLP-1 (min·nM)			
** **OGTT	0.5±0.3	0.4±0.2	0.5±0.4
** **IIGI	0.1±0.1	0.1±0.2	0.03±0.1
** *P*** (OGTT vs IIGI)	<0.01	<0.05	<0.01
⊿AUC-Glucagon 0-180min (min·nM)			
** **OGTT	-958±833	-1624±1119	-397±554
** **IIGI	-760±572	-1727±1269	-784±359
** *P*** (OGTT vs IIGI)	ns	ns	ns
⊿AUC-Glucagon 0-60min (min·nM)			
** **OGTT	-269±217	-197±410	32±126 *
** **IIGI	-249±150	-389±264	-143±88
** *P*** (OGTT vs IIGI)	ns	ns	<0.05

Area under the curve (AUC) of plasma total GIP, active GIP, total GLP-1, and active GLP-1 concentrations during 75g oral glucose tolerance test (OGTT) and isoglycemic intravenous glucose infusion (IIGI). The change in the plasma glucagon response (⊿AUC) is calculated by subtracting the AUC from the baseline value. Data are mean values ± SD. * represent statistical difference vs NGT, respectively *(p < 0.01)*.

'ns' indicates 'not significant' (P>=0.05).

### Incretin effect

**Figure 4:**
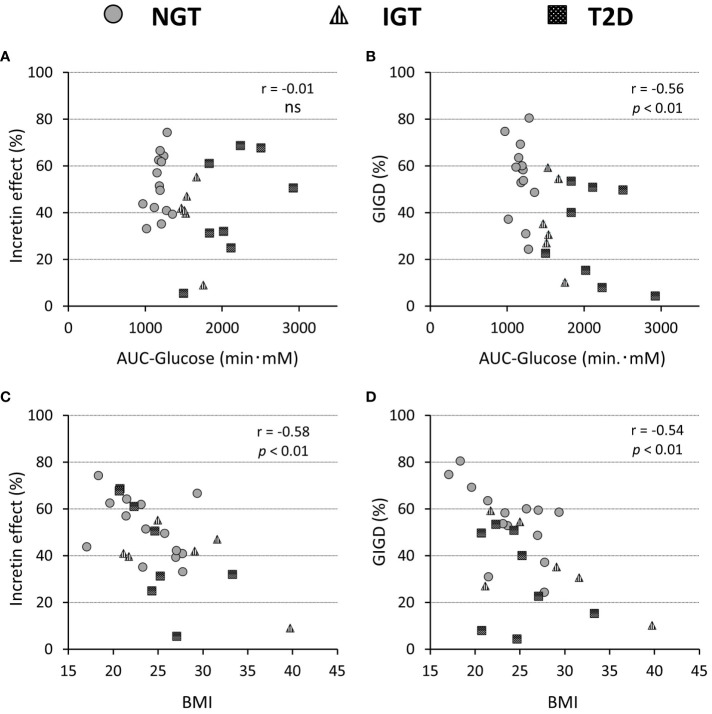
Relationship of the incretin effect calculated by incremental AUC of C-peptide values **(A, C)** and percentage of gastrointestinally mediated glucose disposal (GIGD) **(B, D)**, with the AUC of the glucose concentration during OGTT **(A, B)** and BMI **(C, D)**. Pearson’s correlation tests were carried out to calculate the correlation coefficient (r) and p-values. 'ns' indicates 'not significant' (P>=0.05).

The numerical incretin effect was slightly smaller in the glucose intolerance groups (IGT and T2D) than in the NGT group calculated by IRI (NGT 61.9 ± 15.6%, IGT 44.0 ± 30.2%, T2DM 51.8 ± 21.4%) or CPR (NGT 51.5 ± 12.9%, IGT 39.0 ± 15.8%, T2DM 42.6 ± 22.8%), but the difference was not significant among groups. (**Table 2**). Similarly, the numerical incretin effect showed no relation to the AUC of glucose (AUC-glucose (r = -0.01, *p = ns*), whereas it significantly negatively correlated with BMI (r = -0.58, *p = 0.012*; **Figures 4A, C**).

The amount of intravenous glucose needed to obtain isoglycemia for OGTT was greater in the T2DM group than in the NGT group (52.2 ± 15.2 g vs 33.7 ± 11.9 g, *p = 0.012*; **Table 2**). Stratifying the glucose infusion amounts into time intervals, the glucose infusion in the T2DM group was significantly greater than that in the NGT group at 40–100 min (**Figures 1J–L**). GIGD calculated from the amount of glucose administered was significantly lower in the T2DM group than in the NGT group (30.4 ± 20.3% vs 55.1 ± 15.8%, *p = 0.012*), and marginally lower in the IGT group than in the NGT group (36.1 ± 18.2% vs 55.1 ± 15.8%, *p = 0.09*; **Figures 1J–L**; **Table 2**). GIGD significantly negatively correlated with both the AUC-glucose (r = -0.56, *p = 0.002*) and BMI (r = -0.54, *p = 0.003*; **Figures 4B, D**).

**Figure 5:**
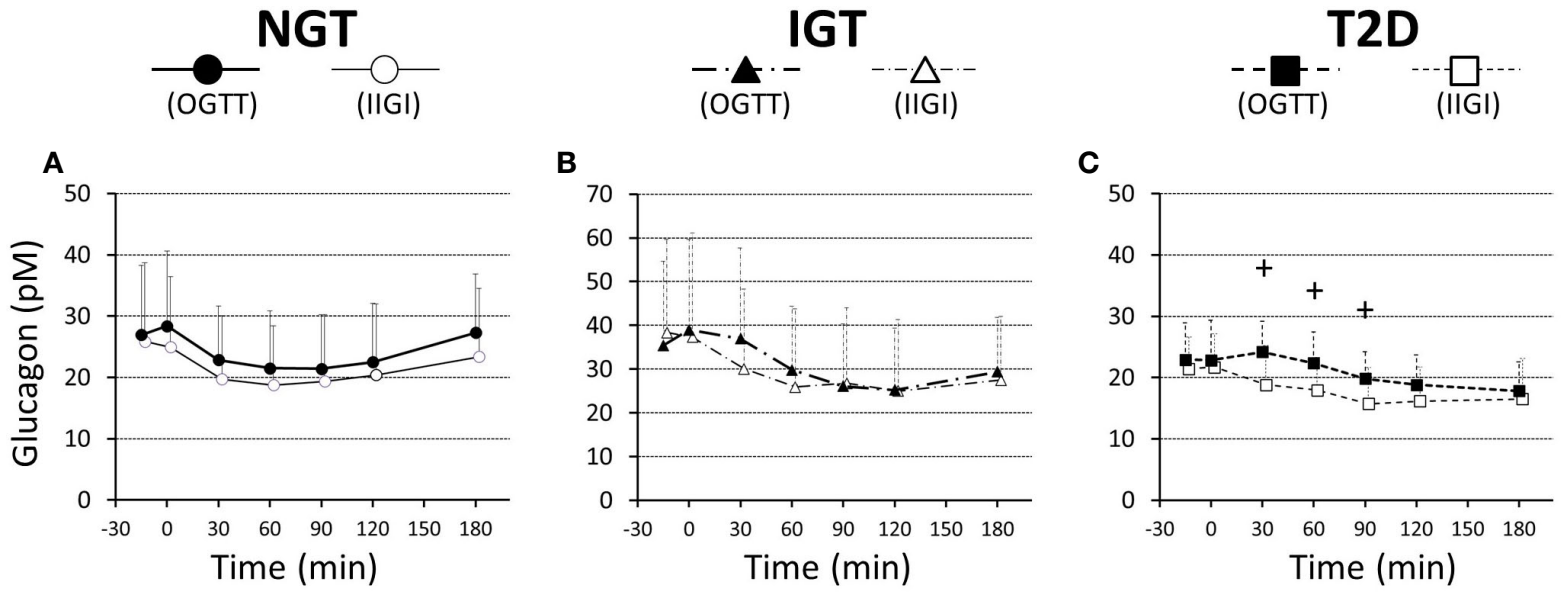
Ratios of plasma glucagon values to the basal values during a 75-g OGTT (filled symbols) and isoglycemic intravenous glucose infusion (open symbols), in participants with NGT **(A)**, IGT **(B)**, and T2D **(C)**. Data are mean values ± SD. ^+^; *p* < 0.05 vs IIGI.

**Figure 6:**
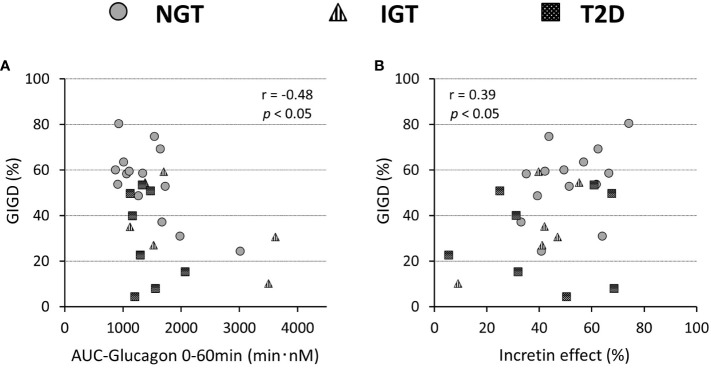
Relationship of the percentage of gastrointestinally-mediated glucose disposal (GIGD) with the incretin effect calculated by the incremental AUC of C-peptide values **(A)** and AUC(0-60min) of the plasma glucagon concentration during OGTT **(B)**. Pearson’s correlation tests were carried out to calculate the correlation coefficient (r) and p-values.

Glucagon suppression after OGTT and IIGT was similar in the NGT and IGT groups. The glucagon concentrations in the T2D group, however, were significantly higher in OGTT than in IIGI, especially in early the phase after glucose loading (**Figure 5**; **Table 3**). AUC-glucagon for 60 min during oral loading was higher in the T2D group than in the NGT group (**Table 3**). Both fasting glucagon (r = 0.47, *p = 0.011*) and AUC-glucagon for 180 min (r = 0.47, *p = 0.013*) significantly correlated with the BMI. Regarding the contribution of glucagon kinetics to the glucose handling capacity, the AUC-glucagon for 60 min subtracted by baseline (i.e., the early post-loading period) negatively correlated with GIGD (r = -0.48, *p = 0.01*; **Figure 6A**). Furthermore, the numerical incretin effect also weakly, but significantly positively correlated with GIGD (r = 0.39, *p = 0.042*; **Figure 6B**).

## Discussion

In the present study, we analyzed participants exhibiting a glucose tolerance spectrum from NGT to early-stage mild T2D. In the T2D participants, despite being in the early stages, we observed a significant reduction in the insulinogenic index, reflecting distinct characteristics observed in Japanese T2D patients (37, 38). This study, therefore, enabled us to investigate the effects of incretin in East Asians, characterized by lower insulin secretion, in comparison with previous findings in Caucasians. Although, the incretin effect was slightly smaller in the T2D group than in the NGT group, the difference was not significant in contrast to previous reports in Western Caucasians (6–11). A reduced numerical incretin effect, however, was clearly observed in clear with an increase in BMI.

Several studies in Caucasians report decreases in the numerical incretin effect in response to impaired glucose tolerance (6–11), but in Koreans, no significant differences in the numerical incretin effect between those with NGT and T2D are reported (21). Our findings arevealed that the decrease in glucose tolerance up to early T2D is not always parallel with the decrease in the incretin effect in contrast to that in Caucasians. These characteristics of the incretin effect are common to East Asians, suggesting a potential racial difference in the action of incretin (12). IGT is reported to decrease the incretin effect in Caucasians (8). Our results demonstrated no clear difference in the numerical incretin effect in Japanese with IGT compared to those with NGT and T2D.

Here, we consider the insulin secretory response separately after oral glucose loading, the insulin secretory response after intravenous loading, and the difference between these two conditions as the intestinal factor-derived insulin secretory response (IdIR) (**Figure 2**). Along with the diminished insulin secretory response to direct (intravenous) glucose loading in the case of β-cell dysfunction during impaired glucose tolerance, the IdIR is also decreased (39). Detailed observations of insulin secretion in previous reports (7, 8) revealed that the reduction of the IdIR ratio to the insulin secretory response to intravenous glucose loading along with glucose intolerance is more prominent in Caucasians than in Japanese subjects (**Figures 2A–C**). For this reason, the reduction of the numerical incretin effect in Japanese with T2D is not so large. Incretin secretion was not affected by differences in glucose tolerance, consistent with previous reports (40–44). In terms of incretin action, a recent genome-wide association study (45) reported that Japanese have a higher proportion of a genetic GLP-1 receptor variant that is associated with greater insulin secretion induced by GLP-1 stimulation (46). This background might contribute to the relatively preserved IdIR compared with the strongly diminished insulin response to intravenous glucose loading in our results. Furthermore, this may be why the effectiveness of incretin-based therapy for T2D is more apparent in Asians (47, 48).

The numerical incretin effect decreased as BMI increase. As BMI increases, the insulin secretory response to intravenous glucose loading increases, but IdIR was not enhanced (**Figures 2H, I**), resulting in a diminished numerical incretin effect. The pattern is consistent with finding from previous studies on insulin secretion in Caucasians (8, 10). These findings may relate to the greater effectiveness of incretin-based therapies for patients with T2D with relatively lower BMI (47–49).

The GIGD can be considered as an index of the capacity of additional oral glucose loading under the blood glucose excursion observed in intravenous loading (12, 25). In this study, GIGD decreased as glucose tolerance deteriorated, which is consistent with previous studies in Caucasians (6, 7, 9, 10) and East Asians (21). In the T2D group, suppression of glucagon secretion was insufficient after oral glucose loading compared with after intravenous glucose loading, as previously reported (50, 51). GIGD negatively correlated with the AUC-glucagon in OGTT, suggesting that GIGD reflects the role of glucagon in glucose excursions (20, 52, 53). Besides glucagon, GIGD weakly, but significantly, positively correlated with the incretin effect calculated by CPR in the present study. We defined GIGD as representing an integrated and more accurate incretin effect including the secretion and action of both insulin and glucagon (12, 25, 54, 55). GIGD also decreased with an increase in the BMI, which was accompanied by increasing glucagon concentrations. Increased glucagon secretion in obesity is also reported in Caucasians (10, 56). The dysregulated of glucose metabolism in obesity results from by both the inadequate increase in insulin secretion through the numerical incretin effect and insufficient suppression of glucagon secretion. On the basis of the findings to date, this mechanism may be common between Caucasian and East Asian populations.

Our study has some limitations. First, the glucose tolerance groups were not matched for age. Generally, β-cell function decreases with age. Our finding that the incretin effect was not diminished in older subjects with impaired glucose tolerance supports the notion that the numerical incretin effects differ from those in Caucasians, but an age-matched analysis may be necessary for confirmation. Second, the gastric emptying rate was not measured. A decrease in the gastric excretion rate due to impaired glucose tolerance affects incretin secretion and the evaluation of its effect. It is reported that the excretion rate decreases with an increase in the glucose load but that there is no significant difference among different glucose tolerance groups with a 75-g glucose load (9). This may not be applicable in the East Asian population. Third, this study was conducted with a small number of subjects in a single facility and there were more male than female participants. To clarify the different characteristics between Asian and Caucasian populations, a large-scale multi-center study is needed.

In conclusion, in Japanese, obesity has a more pronounced impact on reducing the numerical incretin effect compared with impaired glucose tolerance. On the other hand, GIGD as the integrated glucose handling capacity encompassing the glucagon inhibitory effect decreases with worsening glucose intolerance and increasing obesity, which may be a common pathophysiologic characteristic between Asians and Caucasians. A detailed examination of the incretin effect by taking into account the characteristics of each race is critically important for elucidating the pathogenesis of diabetes, which will lead to more appropriate therapeutic approaches tailored to each condition.

## Data availability statement

The raw data supporting the conclusions of this article will be made available by the authors, upon reasonable request.

## Ethics statement

The studies involving humans were approved by the ethics committee of Kyoto University. The studies were conducted in accordance with the local legislation and institutional requirements. Written informed consent for participation in this study was obtained from all participants.

## Author contributions

AH: Writing – original draft, Writing – review & editing, Investigation, Conceptualization, Resources, Data curation, Formal analysis, Methodology, Project administration. NH: Writing – review & editing, Investigation, Conceptualization. AM: Writing – review & editing, Investigation, Resources, Methodology. SY: Writing – review & editing, Investigation. EJ: Writing – review & editing, Investigation. KS: Writing – review & editing, Investigation. NI: Writing – review & editing, Conceptualization, Supervision.
